# Causal effects of endometriosis stages and locations on menstruation, ovulation, reproductive function, and delivery modes: a two-sample Mendelian randomization study

**DOI:** 10.3389/fendo.2024.1328403

**Published:** 2024-08-02

**Authors:** Lin Shen, Jie Li, Hanwang Zhang, Yiqing Zhao

**Affiliations:** ^1^ Department of Reproductive Medicine, Tongji Hospital, Tongji Medical College, Huazhong University of Science and Technology, Wuhan, China; ^2^ National Research Institute for Family Planning, Beijing, China; ^3^ Graduate School, Chinese Academy of Medical Sciences and Peking Union Medical College, Beijing, China

**Keywords:** endometriosis, menstruation, ovulation, reproductive function, mode of delivery, Mendelian randomization

## Abstract

**Background:**

Endometriosis is a chronic inflammatory disease of women during their reproductive years. The relationship between the severity and location of endometriosis and menstruation, ovulation, reproductive function, and mode of delivery remains unclear.

**Methods:**

We explored the association between the various phenotypes of endometriosis and menstruation, ovulation, reproductive function, and mode of delivery, using two-sample Mendelian randomization (MR) and summary data on endometriosis stages and locations from the FinnGen consortium and women’s menstruation, ovulation, reproductive function, and mode of delivery from OpenGWAS and ReproGen. Inverse-variance weighting was used for the primary MR analysis. In addition, a series of sensitivity analyses, confounding analyses, co-localization analyses, and multivariate MR analyses were performed.

**Results:**

MR analysis showed a negative effect of moderate to severe endometriosis on age at last live birth (OR = 0.973, 95% CI: 0.960–0.986) and normal delivery (OR = 0.999, 95% CI: 0.998–1.000; values for endpoint were excluded), ovarian endometriosis on age at last live birth (OR = 0.976, 95% CI: 0.965–0.988) and normal delivery (OR = 0.999, 95% CI: 0.998–1.000; values for endpoint were excluded), and fallopian tubal endometriosis on excessive irregular menstruation (OR = 0.966, 95% CI: 0.942–0.990). Bidirectional MR analysis showed that age at menarche had a negative causal effect on intestinal endometriosis (OR = 0.417, 95% CI: 0.216–0.804). All MR analyses were confirmed by sensitivity analyses, and only the genetic effects of moderate to severe endometriosis on normal delivery and age at last live birth were supported by co-localization evidence.

**Conclusion:**

Our findings deepen the understanding of the relationship between various types of endometriosis and menstruation, ovulation, reproductive function, and mode of delivery and clarify the important role of moderate to severe endometriosis.

## Introduction

Endometriosis is a chronic inflammatory disease that primarily affects women of reproductive age, with a prevalence of up to 10% (1, 2). It is characterized by the presence of endometrial tissue outside the body of the uterus and is associated with pelvic pain, dysmenorrhea, and infertility and its treatment is limited to hormonal therapy or surgical removal of disease (1, 3). Endometriosis is typically staged according to the revised American Society of Reproductive Medicine (ASRM) criteria, with milder lesions in stages 1–2 and more severe lesions in stages 3–4. In addition, ectopic endometrium can invade almost any part of the body, including the lungs and pleura, but most commonly the pelvic organs and parietal peritoneum (4).

The etiology of endometriosis remains unknown, but several risk factors have been reported. To date, it is known that earlier age at menarche and shorter menstrual cycles are associated with an increased risk of endometriosis, whereas higher parity is associated with a lower risk (5). However, these studies have lacked associations between various phenotypic features of endometriosis, including ASRM stages and locations, and menstruation, ovulation, reproductive function, and mode of delivery, consistently focusing on the broad category of endometriosis, with far less information on its subcategories. Moreover, most of these studies only included risk factors for endometriosis and lacked findings on the possible consequences of endometriosis.

In addition, studies on factors such as menstruation, ovulation, reproductive function, and mode of delivery in relation to endometriosis have been mostly limited to epidemiologic observations and cohort studies. Therefore, it is difficult to draw causal conclusions from these studies due to possible biases and potential confounders. In this manuscript, we investigated the risk factors and consequences of endometriosis based on GWAS using Mendelian randomization (MR). MR is an analytical method for assessing causal inference in epidemiologic studies in which endometriosis can be either an exposure or an outcome, and uses genetic variants that are strongly associated with exposure as instrumental variables (IVs) to assess the causal relationship between exposure and outcome.

Here, we reported a comprehensive GWAS-based MR analysis of the association between different stages and locations of endometriosis and menstruation, ovulation, reproductive function, and mode of delivery, elucidating the causal effects of endometriosis sub-phenotypes leading to various components of the complex pathophysiology and improving our understanding of the risk factors and consequences of endometriosis.

## Methods

We selected genetic variants that were strongly associated with different ASRM stages and locations of endometriosis from the FinnGen consortium with a sample size of 210,870 women, including endometriosis ASRM stages 1–2 and ASRM stages 3–4, and endometriosis of ovarian, fallopian tube and pelvic peritoneum, rectovaginal septum and vagina, and intestine. At the same time, we collected the summary level data from OpenGWAS and ReproGen study for GWAS on menstruation (excessive irregular menstruation, and menstrual cycle length), ovulation (age at menarche, age at natural menopause, years ovulating), reproductive function (age at first live birth, age at last live birth, number of live births, spontaneous abortion), and mode of delivery (normal delivery, caesarean section) (6, 7). In this study, we used two-sample MR analysis to investigate the genetic basis between different stages and locations of endometriosis and menstruation, ovulation, reproductive function, and mode of delivery and assess the causal effects among them.

### Data information

**Figure 1:**
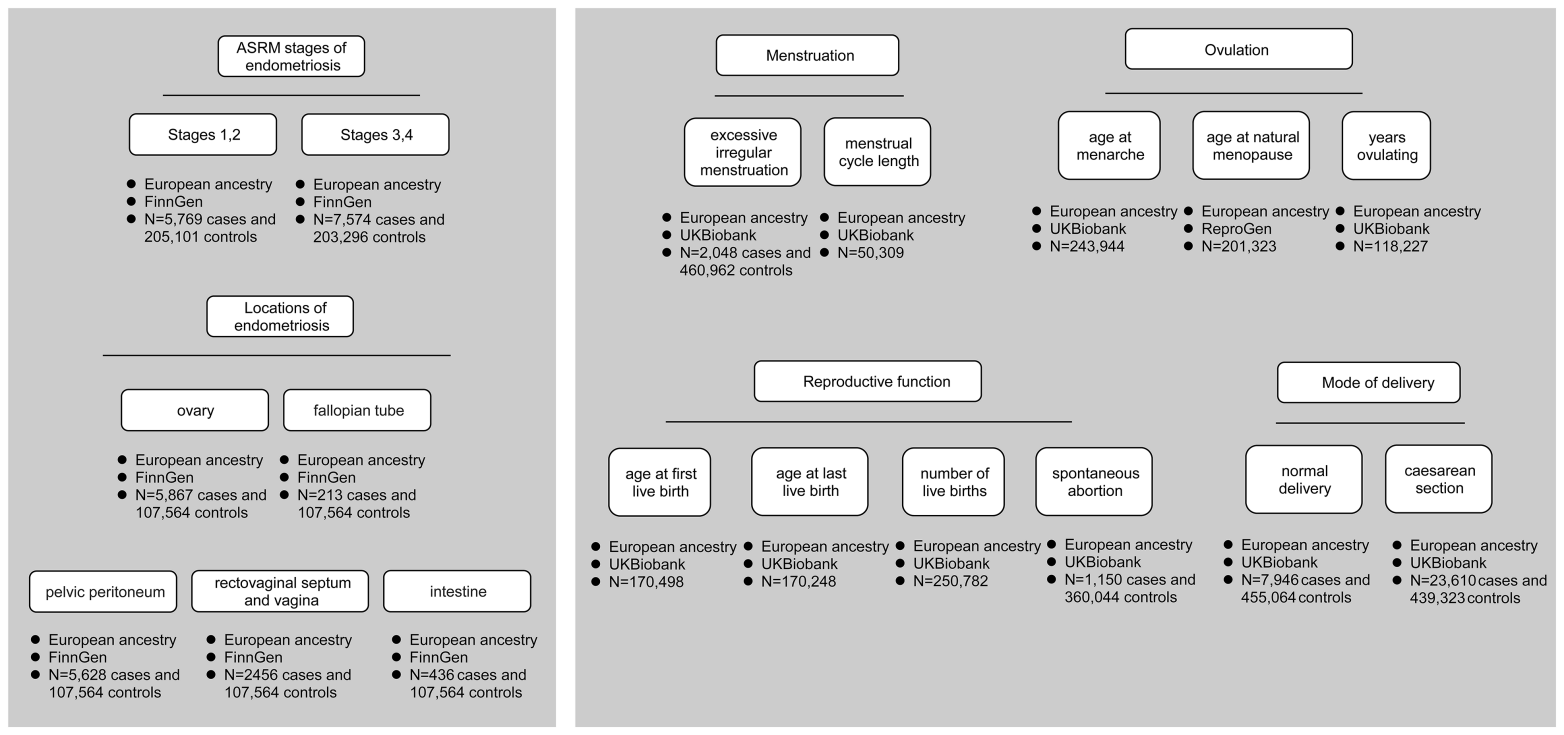
Data overview. It shows different ASRM stages and locations of endometriosis, menstruation, ovulation, reproductive function, and mode of delivery. Each phenotype labels the data source and the number of cases and controls.

Phenotypes were described in detail as follows: (1) different ASRM stages and locations of endometriosis; (2) menstruation (excessive irregular menstruation and menstrual cycle length); (3) ovulation (age at menarche, age at natural menopause, and years ovulating); (4) reproductive function (age at first live birth, age at last live birth, number of live births, and spontaneous abortion); (5) mode of delivery (normal delivery and caesarean section). All genetic association estimates were derived from GWAS studies conducted in European populations, and the design and analysis protocols (such as adjustment for covariates) were available for query in publications (7–9). An overview of the data is shown in **Figure 1**, and detailed information can be found in **Additional File 2: Table 1**.

#### Endometriosis

In FinnGen, N14 codes for endometriosis. The subitems of N14 were obtained, which were ASRM stages 1–2 and ASRM stages 3–4, as well as endometriosis of ovary, fallopian tube, pelvic peritoneum, rectovaginal septum and vagina, and intestine. Controls for a particular phenotype of endometriosis were those individuals who were not cases.

#### Menstruation

In OpenGWAS, ICD10: N92.1 was included in the analysis, and women with this code were diagnosed with excessive and frequent menstruation with irregular cycles (N = 2,048). It is referred to in this manuscript as excessive irregular menstruation. The length of menstrual cycle refers to the time since the last menstrual period (N = 50,309).

#### Ovulation

A total of 243,944 individuals of European ancestry who self-reported their age at menarche, ranging from 5 to 25 years, were included in OpenGWAS. Summary data on the age at natural menopause were reported by Ruth et al., who reported an age of 40 to 60 years, and were stored in the ReproGen database (N = 201,323) (8). Years ovulating was defined as the time between age at menarche and menopause, calculated by D’Urso et al. using UK Biobank data, as detailed in the publication (10).

#### Reproductive function

Age at first and last live birth was collected from women who reported having more than one child. The number of live births ranged from 0 to 22, with a median of 2. ICD10: O03 coded for spontaneous abortion.

#### Mode of delivery

GWAS summary data on normal deliveries and caesarean sections were available in OpenGWAS, which included 7,946 normal deliveries and 23,610 caesarean sections.

### Selection of genetic instruments and data harmonization

We selected all single-nucleotide polymorphisms (SNPs) strongly associated with exposure as instrumental variables (IVs), with a genome-wide significance threshold of *p* < 5 × 10^−8^, which was *p* < 5 × 10^−6^ for endometriosis of the fallopian tube and intestine. Moreover, the strength of each IV was assessed, and when F >10, the SNP was included (10). All SNPs in linkage disequilibrium (LD) were trimmed with a window of r^2^ < 0·001 and size <10,000 kb, and at the same time, SNPs with minor allele frequency (MAF) >0.5% were retained. IVs that met the above quality control are shown in **Additional File 1: Table 1**. Then, SNP exposure and SNP outcome were harmonized with the use of the “harmonise_data” function (11). SNPs for incompatible alleles and being palindromic with intermediate allele frequencies were removed during harmonization, if present.

### Mendelian randomization analysis

Univariate MR analyses were performed to estimate the causal effect of each type of endometriosis on (1) menstruation, (2) ovulation, (3) reproductive function, and (4) mode of delivery. Inverse variance weighted (IVW) MR was used as the primary analysis method unless only one SNP was available (in which case the Wald ratio was used). IVW can provide unbiased estimates of outcomes in the absence of horizontal pleiotropy in IVs (12). In addition, the IVW method can account for the heterogeneity of causal estimates obtained from individual variants (13, 14).

### Bidirectional Mendelian randomization analysis

To investigate the possibility of reverse causality between exposure and outcome, bidirectional MR analyses were performed (exposures and outcomes were reversed in the analyses). We used a *p* < 5 × 10^−8^ to select the IVs associated with exposure, and the *p* value threshold was appropriately relaxed to 5 × 10^−6^ for excessive irregular menstruation, menstrual cycle length, spontaneous abortion, normal delivery, and cesarean section. Other analysis details were the same as for the MR analysis. The available IVs are shown in **Additional File 1: Table 2**.

### Sensitivity analyses

For each causal effect detected, we used a series of sensitivity analyses to assess the robustness of the MR analysis. First, Cochran’s Q statistic, including both IVW and MR–Egger methods, was used to evaluate the heterogeneity of IVs in the causal effects. Second, MR–Egger regression was used to evaluate the presence of horizontal pleiotropy; if the intercept term equals zero, it indicates that horizontal pleiotropy does not exist. Third, the MR‐pleiotropy residual sum and outlier (MR-PRESSO) analysis obtained horizontal pleiotropy by detecting abnormal IVs in causal effects. If abnormal IVs were present, outliers were removed, and MR analyses and sensitivity analyses were repeated. When <50% of the instruments showed horizontal pleiotropy, the MR-PRESSO test was most appropriate (15). Finally, we performed a leave-one-out sensitivity analysis by stepwise elimination of each IV to see if the results changed after each SNP was removed.

### Confounding analysis

We used the “phenoscanner” package (version 1.0) for confounding analysis (16). Diseases/physical conditions significantly associated with IV were explored using *p* < 5 × 10^−8^ as the threshold.

### Colocalization analysis

The coloc R package (version 5.2.2) was used to perform a Bayesian test for co-localization of the two traits to estimate the posterior probability of shared variation (17). We retrieved all SNPs within the upper and lower 50 kb of each IV for co-localization analysis to analyze the posterior probability of H4 (PP.H4), with PP.H4 >0.95 indicating co-localization of the two traits.

### Multivariable Mendelian randomization

The exposures included in the multivariable MR (MVMR) analysis were ASRM stages 3–4 and ovarian endometriosis. Univariate MR analysis suggested a causal relationship between these exposures and age at last live birth and normal delivery. We used MVMR to account for potential horizontal pleiotropy (18). The inclusion of IVs for MVMR was the same as for MR analysis.

### Statistical analysis

The results of MR analysis were presented as odds ratio (OR) and 95% confidence interval (CI). Due to the presence of multiple comparisons with causal effects, we corrected the *p*-values with the false discovery rate (FDR), with a threshold of an adjusted *p*-value < 0.1. This work was performed under R packages “TwoSampleMR” (version 0.5.7), “MR-PRESSO” (version 1.0), and “MVMR” (version 0.4) (11, 15).

## Results

### Causal effects of endometriosis stage and location on menstruation, ovulation, reproductive function, and mode of delivery

**Figure 2:**
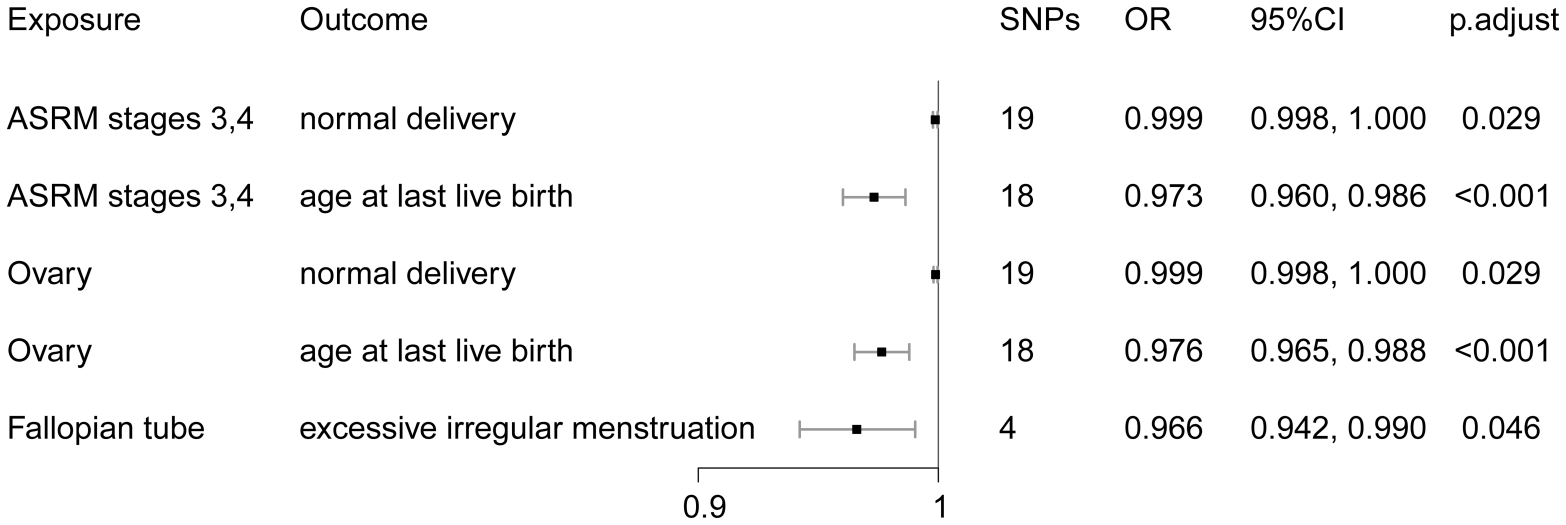
MR estimates for the association between endometriosis stages and locations and menstruation, ovulation, reproductive function, and mode of delivery.

As shown in **Figure 2**, MR analysis showed a negative effect of ASRM stages 3–4 endometriosis on age at last live birth (OR = 0.973, 95% CI: 0.960–0.986) and normal delivery (OR = 0.999, 95% CI: 0.998–1.000, excluding the value of the endpoint 1.000). We also assessed the effect of different locations of endometriosis on various outcomes. IVW estimates showed a negative effect of ovarian endometriosis on age at last live birth (OR = 0.976, 95% CI: 0.965–0.988) and normal delivery (OR = 0.999, 95% CI: 0.998–1.000, values for endpoint 1.000 were excluded). Endometriosis in the fallopian tube was also observed to have a negative effect on excessive irregular menstruation (OR = 0.966, 95% CI: 0.942–0.990) (**Figure 2**). All MR estimates can be viewed in **Additional File 1: Table 3**.

Horizontal pleiotropy and heterogeneity analyses showed that the causal effects of ASRM stages 3–4 endometriosis and ovarian endometriosis on normal labor and tubal on excessive menstruation were robust (**Additional File 2: Tables 2, 4, 6**). MR-PRESSO analyses showed significant horizontal pleiotropy between IVs in the causal effect of ASRM stages 3–4 endometriosis and ovarian endometriosis on age at last live birth, identifying three abnormal IVs, respectively (**Additional File 2: Tables 3, 5**). After removing the outliers, the MR analysis was re-performed, and horizontal pleiotropy and heterogeneity between IVs were corrected (**Additional File 2: Tables 3, 5**). The leave-one-out test did not identify SNPs with abnormal effects (**Additional File 2: Figure 1**). This manuscript only listed the corrected MR Results (the same below).

### Bidirectional Mendelian randomization analysis

**Figure 3:**
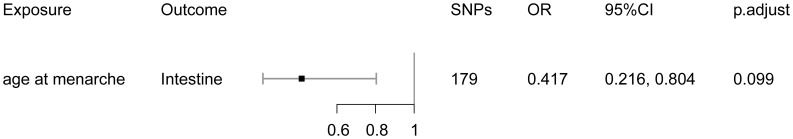
Bidirectional MR analysis.

Bidirectional MR analysis showed that age at menarche had a negative causal effect on intestinal endometriosis (OR = 0.417, 95% CI: 0.216–0.804), with an increase in age at menarche and a decrease in risk of the disease (**Figure 3**). No other causal effects were detected. **Additional File 1: Table 4** demonstrated all MR estimates.

There was horizontal pleiotropy in the IVs for age at menarche; MR-PRESSO analysis excluded one SNP (**Additional File 2: Table 7**). After removal of outliers, horizontal pleiotropy of IVs was corrected, whereas heterogeneity among IVs remained (**Additional File 2: Table 7**). However, our results were generated based on the random-effects model (IVW), which remained robust in the presence of heterogeneity (13, 14). The leave-one-out test showed no outliers in the IVs (**Additional File 2: Figure 1**).

### Confounding analysis

IVs in causal effects were summarized and analyzed with various confounders by Phenoscanner. We found that potential confounders of ASRM stages 3–4 and ovarian endometriosis mainly included trunk predicted mass, birth weight, coagulation dysfunction, height, heel bone mineral density, atopic dermatitis, potential confounders of fallopian tubal endometriosis mainly including mean plasma volume, mean hemoglobin, and erythrocyte distribution width, and potential confounders of age at menarche mainly including body mass index, birth weight, and triglycerides (**Additional File 2: Figures 2, 3, 4, 5**).

### Colocalization analysis

By co-localization analysis (PP.H4 >0.95), we found evidence to support the existence of shared causal variants between ASRM stages 3 and 4 endometriosis with normal delivery and age at last live birth (**Additional File 2: Table 8**). There was no evidence of co-localization for the remaining four causal effects.

### Multivariable Mendelian randomization

We further assessed the association between ASRM stages 3–4 and ovarian endometriosis with normal delivery and age at last live birth. Both ASRM stages 3–4 and ovarian endometriosis were not associated with normal delivery and age at last live birth in the MVMR framework (**Additional File 2: Tables 9**, **10**).

## Discussion

In this study, we performed a two-sample MR analysis using large genome-wide association datasets to evaluate putative causal relationships between seven types of endometriosis and menstruation, ovulation, reproductive function, and mode of delivery. We found negative causal effects of ASRM stage 3–4 endometriosis on age at last live birth and on normal delivery, ovarian endometriosis on age at last live birth, and normal delivery, and fallopian tubal endometriosis on excessive irregular menstruation. In addition, bidirectional MR showed that age at menarche had a negative causal effect on intestinal endometriosis. All MR analyses were confirmed by sensitivity analyses, and further, the genetic effects of ASRM stage 3–4 endometriosis on normal delivery and age at last live birth were supported by co-localization evidence. Our findings highlight the genetic association between stages and locations of endometriosis and menstruation, ovulation, reproductive function, and mode of delivery.

Previous efforts, including cohort studies and observational studies, have focused on the effect of the severity of endometriosis on the ability of assisted reproductive technology (ART) to achieve fertility (19–22). We reported an association between endometriosis severity and normal delivery and age at last live birth, with moderate-to-severe endometriosis impairing normal delivery and advancing the age at last live birth. Moreover, our findings supported that menstruation, ovulation, reproductive function, and mode of delivery were not associated with mild endometriosis.

We also investigated the causal effects of endometriosis locations on menstruation, ovulation, reproductive function, and mode of delivery. In this study, our analyses found that ovarian endometriosis showed a greater impact on women, followed by fallopian tube, and endometriosis of pelvic peritoneum, rectovaginal septum and vagina, and intestine were unrelated exposures. In brief, ovarian endometriosis brought forward the age at last live birth and impaired normal delivery, whereas fallopian tubal endometriosis reduced the risk of excessive irregular menstruation.

The association between ovarian endometriosis and age at first live birth was noted by Tuominen et al. They found that the age at first live birth was greatest in women with ovarian endometriosis compared with those with peritoneal and deep endometriosis (23). This is inconsistent with our finding that we did not find such a correlation. First, we compared ovarian endometriosis with non-endometriosis and endometriosis elsewhere, whereas Tuominen et al. compared ovarian endometriosis with peritoneal and deep endometriosis. Second, our study included a more general population, whereas the study by Tuominen et al. was limited to the first live birth before surgical confirmation of endometriosis and excluded women with mild symptoms who were conservatively diagnosed and treated for endometriosis. In addition, Parazzini et al. also suggested that age at first live birth was not associated with endometriosis (24).

Menstrual cycle length has been widely reported to be associated with endometriosis (5). Short menstrual cycles increase the risk of endometriosis (25, 26). However, we did not find an association between menstrual cycle length and endometriosis. In fact, the effect of the menstrual cycle length on endometriosis is inconsistent across studies, and several studies suggested that the menstrual cycle length was not related to endometriosis (27, 28). Similarly, we did not find an association between endometriosis and the number of live births, although it was previously thought that the risk of endometriosis decreased with increasing number of births (24, 28, 29).

In addition to menstrual cycle length, age at menarche has also been reported. The earlier the age at menarche, the higher the risk of endometriosis (25, 30, 31). However, it has also been shown that there was no association between endometriosis and age at menarche (24, 28, 32). We found that the effect of age at menarche on endometriosis may be limited. The earlier the age at menarche, the higher the risk of intestinal endometriosis. This finding may explain the discrepancy between studies.

In addition, negative effects on age at last live birth and normal delivery were observed for both moderate-to-severe endometriosis and ovarian endometriosis. These two types of endometriosis can lead to abnormal delivery and an earlier age at last live birth, shortening reproductive life. This may suggest that clinical attention needs to be paid to the early detection and timely intervention of these two types of endometriosis to ameliorate their adverse effects on women’s labor and reproductive life span.

When both exposures (moderate-to-severe endometriosis and ovarian endometriosis) were considered in the same multivariate model, neither was associated with outcomes, indicating collinearity of moderate-to-severe endometriosis and ovarian endometriosis. In fact, there was substantial overlap between IVs in moderate-to-severe endometriosis and ovarian endometriosis. We speculated that there might be a causal effect between moderate-to-severe endometriosis and ovarian endometriosis, i.e., ovarian endometriosis tends to be more severe. This is beyond the scope of this manuscript, and therefore we did not explore it further, but it could be explored in the future.

The main strength of this study is the utilization of summary-level data on non-overlapping exposures and outcomes in the two-sample MR framework for causal inference. This is a comprehensive analysis of the genetic association between the stages and locations of endometriosis and menstruation, ovulation, reproductive function, and mode of delivery. In addition, we conduct a series of sensitivity analyses, confounding analyses, and co-localization analyses to explore the bias introduced by violating the MR assumption. We also used MVMR to explore specific biases caused by horizontal pleiotropy via moderate-to-severe endometriosis and ovarian endometriosis.

Several limitations of this study should be considered when interpreting the results. First, our findings may have been affected by cohort selection bias, with the population in the sample representing a subset of the total population. In addition, because all genetic variants were derived from genomic studies of European populations, it is not known whether the findings of this study apply to other ethnic populations. Second, endometriosis may occur not only at the five locations described in this manuscript but also at other locations, such as lungs and pleura. We only performed the analyses of the five common locations of endometriosis, and the reason for not analyzing other locations was the lack of corresponding data. Next, due to the lack of IVs, SNPs used for several exposures in the analysis did not meet the traditional GWAS significance threshold (p < 5 × 10^−8^), which was relaxed to <5 × 10^−6^. This did not affect the reliability of the study’s conclusions, and our main conclusions are based on strict thresholds and a series of subsequent analyses.

In conclusion, this two-sample MR study provided a comprehensive analysis of the genetic associations between stages and locations of endometriosis and menstruation, ovulation, reproductive function, and mode of delivery. Our study elucidates that moderate-to-severe endometriosis and ovarian endometriosis can lead to abnormal deliveries and a shortened reproductive life span. Both types of endometriosis should be detected promptly and intervened early in clinical practice.

## Data availability statement

The original contributions presented in the study are included in the article/**Supplementary Material**. Further inquiries can be directed to the corresponding author.

## Ethics statement

Only summary level data were used in this study. Ethical approval is available in the publications (6–8).

## Author contributions

LS: Data curation, Investigation, Validation, Writing – original draft. JL: Methodology, Visualization, Writing – review & editing. HZ: Conceptualization, Writing – review & editing. YZ: Conceptualization, Writing – review & editing.
