# Supplementary material for: Causal effects of endometriosis stages and locations on menstruation, ovulation, reproductive function, and delivery modes: a two-sample Mendelian randomization study
Source: Front Endocrinol (Lausanne). 2024 Aug 2;15:1328403. doi: 10.3389/fendo.2024.1328403 (PMC11327065; doi:10.3389/fendo.2024.1328403)
Supplement: Supplementary file 2 [file DataSheet_2.docx]

Additional file 2

Table 1. Data information.

Table 2-7. The results of tests for horizontal pleiotropy and heterogeneity.

Table 8. Co-location analysis.

Table 9. MVMR analysis (normal delivery as outcome).

Table 10. MVMR analysis (age at last birth as outcome).

Figure 1. The results of the leave-one-out test.

Figure 2. Confounding analysis of ASRM stages 3-4 endometriosis.

Figure 3. Confounding analysis of ovarian endometriosis’

Figure 4. Confounding analysis of fallopian tubal endometriosis.

Figure 5. Confounding analysis of age at menarche.

Table 1. Data information.

| Phenotypes | source | ID (if appliable) |
| --- | --- | --- |
| Excessive irregular menstruation | https://gwas.mrcieu.ac.uk/ | ukb-b-11463 |
| Menstrual cycle length | https://gwas.mrcieu.ac.uk/ | ukb-b-9591 |
| Age at menarche | https://gwas.mrcieu.ac.uk/ | ukb-b-3768 |
| Age at natural menopause | Ruth et al. Nature |  |
| Years ovulating | D'Urso et al. BMC Medicine |  |
| Age at first live birth | https://gwas.mrcieu.ac.uk/ | ukb-b-12405 |
| Age at last live birth | https://gwas.mrcieu.ac.uk/ | ukb-b-8727 |
| Number of live births | https://gwas.mrcieu.ac.uk/ | ukb-b-1209 |
| Spontaneous abortion | https://gwas.mrcieu.ac.uk/ | ukb-b-O03 |
| Normal delivery | https://gwas.mrcieu.ac.uk/ | ukb-b-15084 |
| Caesarean section | https://gwas.mrcieu.ac.uk/ | ukb-b-6863 |
| Endometriosis (all subitems) | https://r9.finngen.fi/ |  |

Table 2. The results of tests for horizontal pleiotropy and heterogeneity.

| Exposure | Outcome | Sensitivity analysis | | |
| --- | --- | --- | --- | --- |
| ASRM stages 3,4 | Normal delivery |  | RSSobs | p value |
|  |  | MR -PRESSO | 14.154 | 0.813 |
|  |  |  |  |  |
|  |  |  | Egger_intercept | p value |
|  |  | MR Egger | 0 | 0.383 |
|  |  |  |  |  |
|  |  |  | Q | p value |
|  |  | MR Egger | 11.858 | 0.809 |
|  |  | IVW | 12.660 | 0.811 |

Table 3. The results of tests for horizontal pleiotropy and heterogeneity.

| Exposure | Outcome | Sensitivity analysis | | | After removing outliers | |
| --- | --- | --- | --- | --- | --- | --- |
| ASRM stages 3,4 | Age at last live birth |  | RSSobs | p value | RSSobs | p value |
|  |  | MR -PRESSO | 52.301 | 0.001 | 28.271 | 0.276 |
|  |  |  |  |  |  |  |
|  |  |  | Egger_intercept | p value | Egger_intercept | p value |
|  |  | MR Egger | 0.004 | 0.900 | 0.004 | 0.228 |
|  |  |  |  |  |  |  |
|  |  |  | Q | p value | Q | p value |
|  |  | MR Egger | 45.172 | 0.001 | 15.536 | 0.486 |
|  |  | IVW | 45.210 | 0.001 | 17.104 | 0.447 |

Outliers: rs10917151, rs11031005, rs17773240.

Table 4. The results of tests for horizontal pleiotropy and heterogeneity.

| Exposure | Outcome | Sensitivity analysis | | |
| --- | --- | --- | --- | --- |
| Ovary | Normal delivery |  | RSSobs | p value |
|  |  | MR -PRESSO | 13.663 | 0.834 |
|  |  |  |  |  |
|  |  |  | Egger_intercept | p value |
|  |  | MR Egger | 0 | 0.647 |
|  |  |  |  |  |
|  |  |  | Q | p value |
|  |  | MR Egger | 12.033 | 0.798 |
|  |  | IVW | 12.250 | 0.834 |

Table 5. The results of tests for horizontal pleiotropy and heterogeneity.

| Exposure | Outcome | Sensitivity analysis | | | After removing outliers | |
| --- | --- | --- | --- | --- | --- | --- |
| Ovary | Age at last live birth |  | RSSobs | p value | RSSobs | p value |
|  |  | MR -PRESSO | 50.039 | <0.001 | 29.749 | 0.231 |
|  |  |  |  |  |  |  |
|  |  |  | Egger_intercept | p value | Egger_intercept | p value |
|  |  | MR Egger | -0.001 | 0.856 | 0.002 | 0.456 |
|  |  |  |  |  |  |  |
|  |  |  | Q | p value | Q | p value |
|  |  | MR Egger | 42.529 | 0.002 | 13.413 | 0.642 |
|  |  | IVW | 42.605 | 0.002 | 13.996 | 0.667 |

Outliers: rs10917151, rs11031005, rs12331471.

Table 6. The results of tests for horizontal pleiotropy and heterogeneity.

| Exposure | Outcome | Sensitivity analysis | | |
| --- | --- | --- | --- | --- |
| Fallopian tube | Excessive irregular menstruation |  | RSSobs | p value |
|  |  | MR -PRESSO | 0.105 | 1 |
|  |  |  |  |  |
|  |  |  | Egger_intercept | p value |
|  |  | MR Egger | 0.033 | 0.889 |
|  |  |  |  |  |
|  |  |  | Q | p value |
|  |  | MR Egger | 0.034 | 0.983 |
|  |  | IVW | 0.059 | 0.996 |

Table 7. The results of tests for horizontal pleiotropy and heterogeneity.

| Exposure | Outcome | Sensitivity analysis | | | After removing outliers | |
| --- | --- | --- | --- | --- | --- | --- |
| Age at menarche | Intestine |  | RSSobs | p value | RSSobs | p value |
|  |  | MR -PRESSO | 245.106 | 0.001 | -12.554 | 0.711 |
|  |  |  |  |  |  |  |
|  |  |  | Egger_intercept | p value | Egger_intercept | p value |
|  |  | MR Egger | -0.012 | 0.563 | -0.003 | 0.869 |
|  |  |  |  |  |  |  |
|  |  |  | Q | p value | Q | p value |
|  |  | MR Egger | 241.984 | 0.001 | 209.882 | 0.046 |
|  |  | IVW | 242.441 | 0.001 | 209.914 | 0.051 |

Outliers: rs9397414.


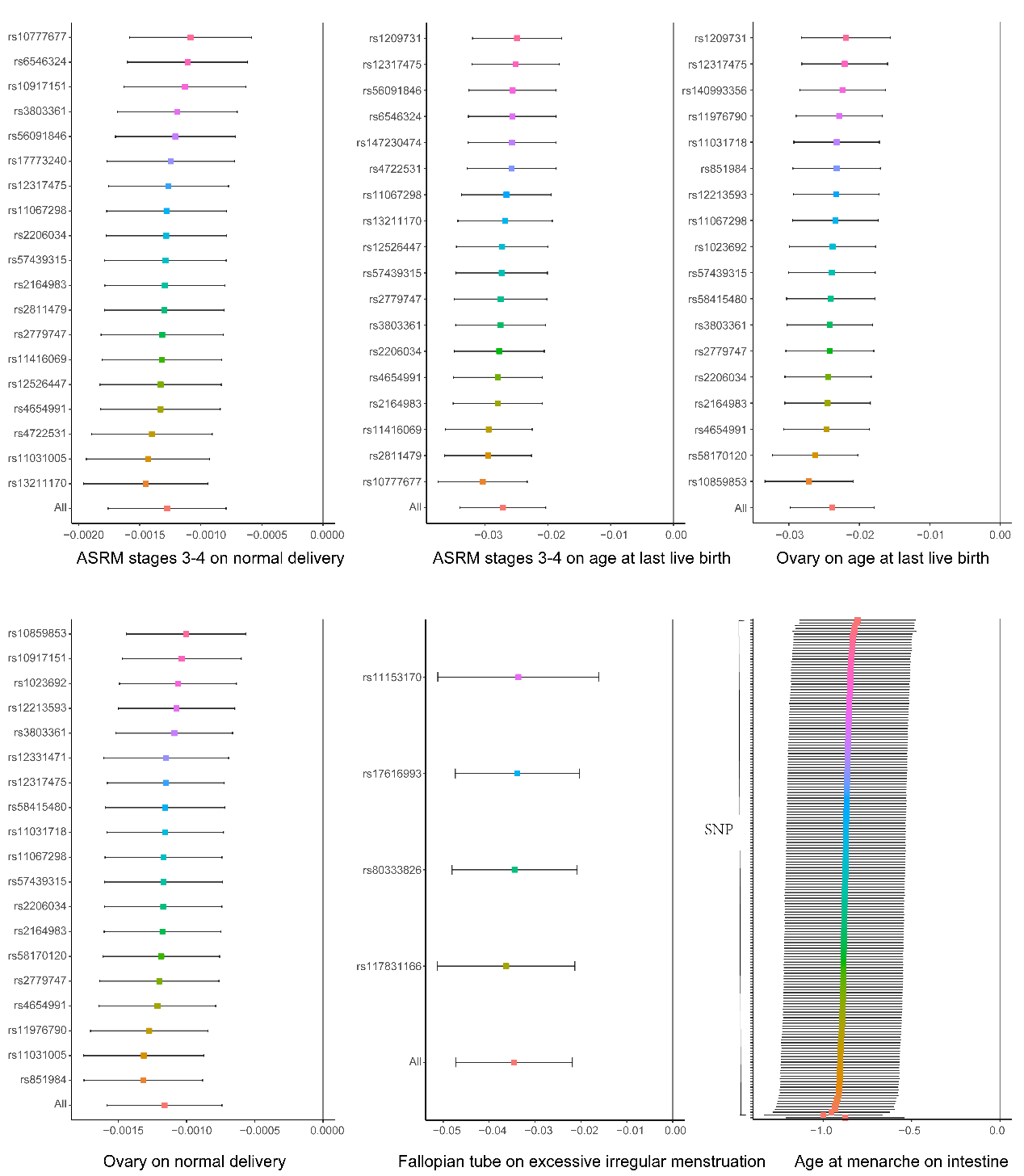
Figure 1. The results of the leave-one-out test.

Figure 2. Confounding analysis of ASRM stages 3-4 endometriosis.


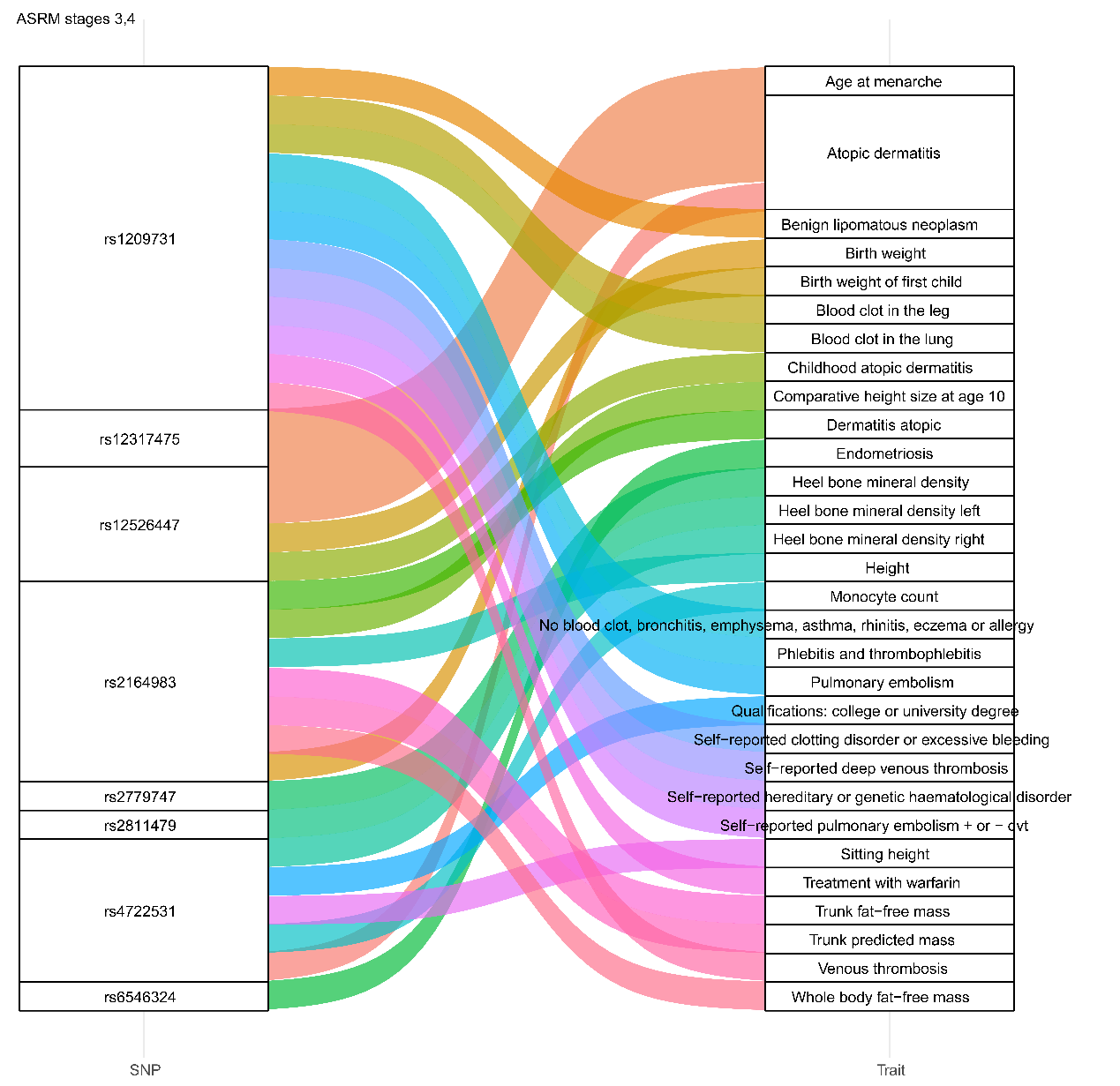


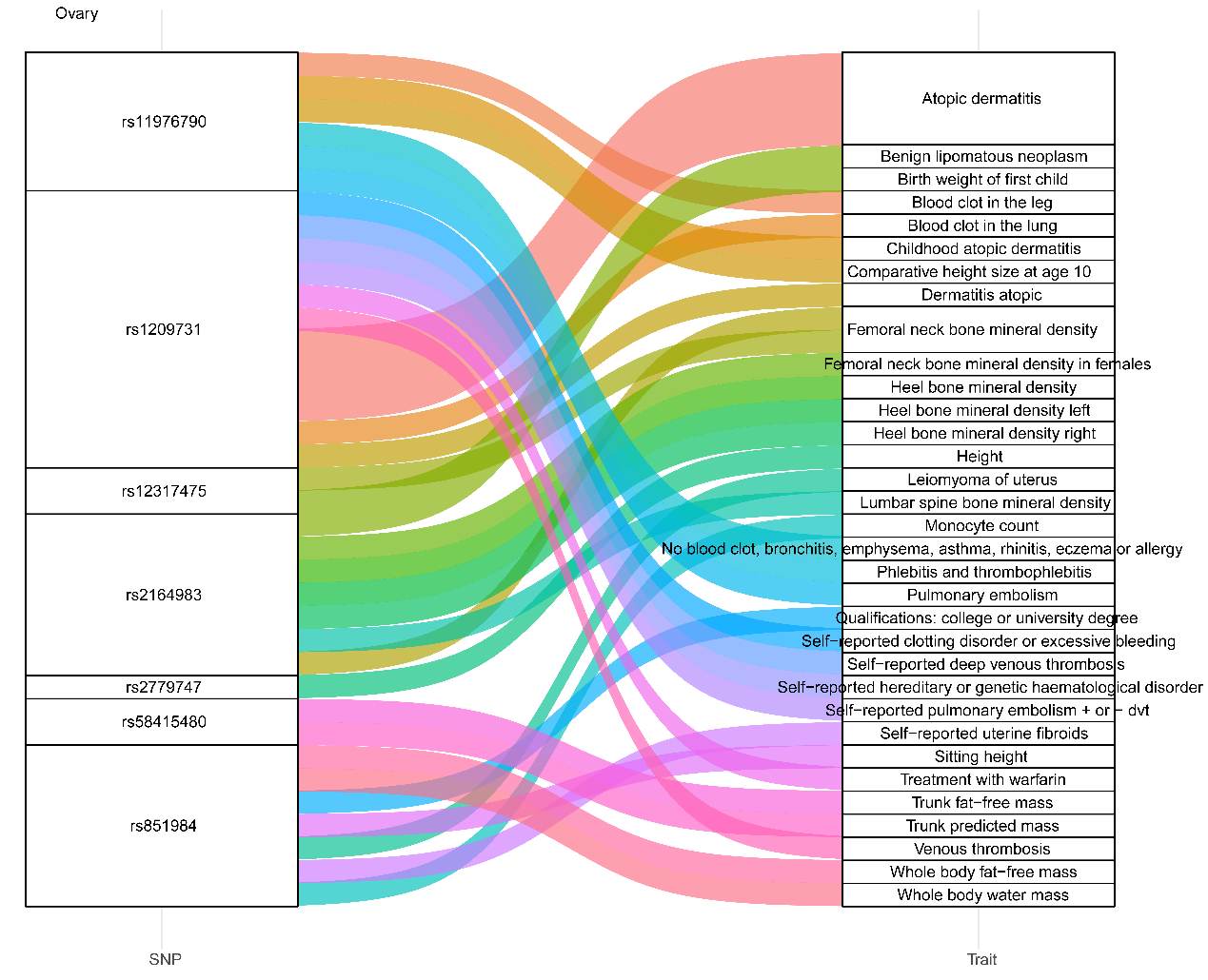
Figure 3. Confounding analysis of ovarian endometriosis.

Figure 4. Confounding analysis of fallopian tubal endometriosis.


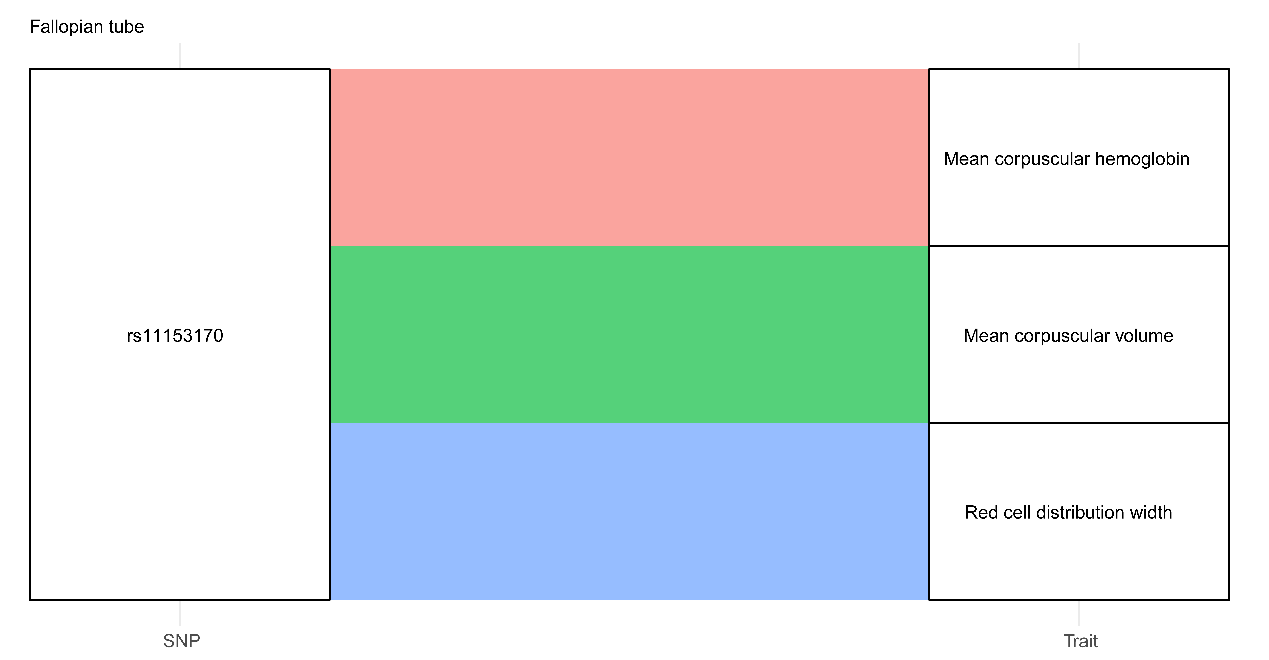


Figure 5. Confounding analysis of age at menarche.
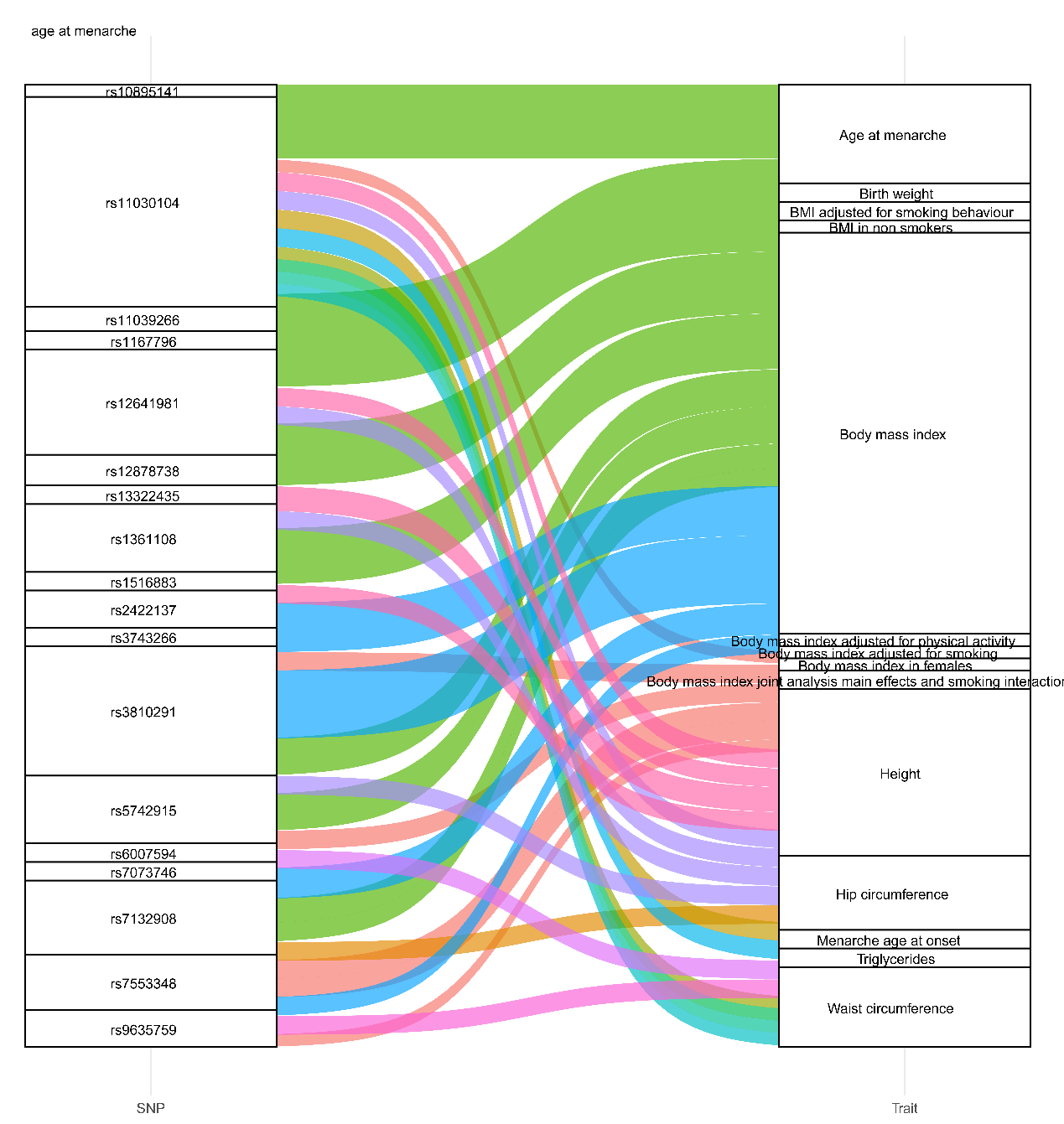


Table 8. Co-location analysis.

| Exposure | Outcome | co-localized locus | PP.H4 |
| --- | --- | --- | --- |
| ASRM stages 3,4 | Age at last live birth | rs13211170 | 0.995 |
| ASRM stages 3,4 | Normal delivery | rs13211170 | 0.971 |

Table 9. MVMR analysis (normal delivery as outcome).

| Exposure | Outcome | MVMR method | p-value |
| --- | --- | --- | --- |
| ASRM stages 3,4 | Normal delivery | IVW | 0.657 |
| Ovary | Normal delivery | IVW | 0.709 |
|  |  |  |  |
| ASRM stages 3,4 | Normal delivery | MR-Egger | 0.786 |
| Ovary | Normal delivery | MR-Egger | 0.775 |
|  |  |  |  |
| ASRM stages 3,4 | Normal delivery | median | 0.923 |
| Ovary | Normal delivery | median | 0.937 |

Table 10. MVMR analysis (age at last birth as outcome).

| Exposure | Outcome | MVMR method | p-value |
| --- | --- | --- | --- |
| ASRM stages 3,4 | Age at last birth | IVW | 0.545 |
| Ovary | Age at last birth | IVW | 0.425 |
|  |  |  |  |
| ASRM stages 3,4 | Age at last birth | MR-Egger | 0.861 |
| Ovary | Age at last birth | MR-Egger | 0.664 |
|  |  |  |  |
| ASRM stages 3,4 | Age at last birth | median | 0.660 |
| Ovary | Age at last birth | median | 0.865 |
